# Clinical characteristics and effects of inhaled corticosteroid in patients with post-COVID-19 chronic cough during the Omicron variant outbreak

**DOI:** 10.1186/s12890-024-02937-7

**Published:** 2024-03-27

**Authors:** Pan-Pan Xie, Yue Zhang, Wen-Kai Niu, Bo Tu, Ning Yang, Yun Fang, Ying-Hui Shi, Fu-Sheng Wang, Xin Yuan

**Affiliations:** 1 Anhui Medical University PLA 307 Clinical College, Beijing, China; 2 Department of Respiratory and Critical Care Diseases, Senior Department of Infectious Diseases, the Fifth Medical Center of PLA General Hospital, Beijing, China; 3 Senior Department of Infectious Diseases, the Fifth Medical Center of PLA General Hospital, National Clinical Research Center for Infectious Diseases, Beijing, China; 4 The Fifth Clinical Medical College of Anhui Medical University, Hefei, Anhui China

**Keywords:** Chronic cough, Inhaled corticosteroid (ICS), Long COVID, Omicron, post-COVID-19 syndrome, Viral infection related-asthma

## Abstract

**Background:**

Chronic cough is a common symptom in patients post the coronavirus disease 2019 (COVID-19). In this study, we aimed to investigate the efficacy of inhaled corticosteroids (ICS) and the clinical characteristics of patients with post-COVID-19 chronic cough during the Omicron era.

**Methods:**

An ambispective, longitudinal cohort study was conducted that included patients with post-COVID-19 who attended the respiratory clinic at our hospital between January 1, 2023, and March 31, 2023 with a complaint of persistent cough lasting more than 8 weeks. At 30 and 60 days after the first clinic visit for post-COVID-19 chronic cough, enrolled patients were prospectively followed up. We compared the changes in symptoms and pulmonary function between patients receiving ICS treatment (ICS group) and those not receiving ICS treatment (NICS group) at the two visits.

**Results:**

A total of 104 patients with post-COVID-19 chronic cough were enrolled in this study (ICS group, *n* = 51; NICS group, *n* = 53). The most common symptoms accompanying post-COVID-19 chronic cough were sputum (58.7%, 61/104) and dyspnea (48.1%, 50/104). Seventy-one (82.6%, 71/86) patients had airway hyperresponsiveness, and 49 patients (47.1%, 49/104) were newly diagnosed with asthma. Most patients (95.2%, 99/104) exhibited improvement at 60 days after the first visit. The pulmonary function parameters of the patients in the ICS group were significantly improved compared to the baseline values (*P* < 0.05), and the improvement in the FEV_1_/FVC was significantly greater than that in the NICS group (*P* = 0.003) after 60 days.

**Conclusions:**

Severe acute respiratory syndrome coronavirus-2 (SARS-CoV-2) may contribute to the pathogenesis of asthma, which could be the underlying cause of persistent cough post-COVID-19 infection. Post-COVID-19 chronic cough during the Omicron era was often accompanied by sputum, dyspnea, and airway hyperresponsiveness. ICS treatment did not have a significant impact on symptom management of post-COVID-19 chronic cough; however, it can improve impaired lung function in in these individuals.

**Supplementary Information:**

The online version contains supplementary material available at 10.1186/s12890-024-02937-7.

## Introduction

The coronavirus disease 2019 (COVID-19) pandemic, caused by severe acute respiratory syndrome coronavirus 2 (SARS-CoV-2), was a public health event with global effects [1, 2]. As of October 25, 2023, the World Health Organization has reported over 770 million cases of SARS-CoV-2 infection, resulting in nearly seven million deaths [3]. The Omicron variant of SARS-CoV-2 was first identified in November 2021 and quickly swept around the world [4]. By the end of 2022, the Omicron variant of SARS-CoV-2 had caused a new round of large-scale epidemics in China. Owing to the ongoing mutation of the virus, numerous people were infected during this wave of the pandemic [5].

Most patients recover completely after an acute infection with SARS-CoV-2; however, a certain percentage of patients experience persistent residual symptoms [6]. Cough is one of the main symptoms in the acute phase of COVID-19, especially when the infection is caused by the Omicron variant [7, 8]. The cough may persist for weeks or months after COVID-19 infection. It has been reported that 20–30% of those infected with SARS-CoV-2 develop a chronic cough, and 2.5% of patients still suffer from coughing 1 year after initial infection [9, 10]. A multicenter observational study of post-discharge conditions in patients who had been hospitalized with COVID-19 reported that 15.4% of patients developed new or worsening cough within 2 months after discharge [11]. Prolonged coughing not only causes distress to patients’ health but also leads to increased stigma and social impact [9].

The mechanism underlying persistent cough after SARS-CoV-2 infection remains unclear. Recently, Chinese experts summarized a consensus on cough after coronavirus infection that proposed symptomatic treatment, with inhaled corticosteroids (ICS) being recommended for patients with airway hyperresponsiveness [12]. However, no study has evaluated the effect of ICS on patients with persistent cough after COVID-19, and there is currently a lack of evidence-based therapeutic regimens for post-COVID-19 chronic cough.

Herein, the study aimed to outline the clinical characteristics and explore the efficacy of ICS of patients with chronic cough after COVID-19 recovery during the Omicron wave.

## Methods

### Participant recruitment

**Fig. 1:**
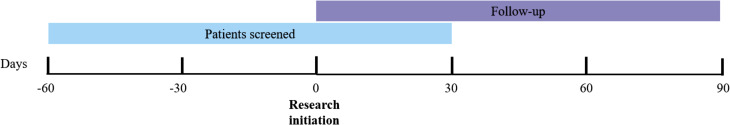
Summary of study design and visits

This ambispective and longitudinal cohort study was designed to investigate the clinical characteristics and effects of ICS therapy in patients with persistent cough lasting more than 8 weeks after SARS-CoV-2 infection. Patients with post-COVID-19 chronic cough were screened between January 1, 2023, and March 31, 2023, at the respiratory clinic of the Fifth Medical Center of the Chinese PLA General Hospital. Eligible subjects were enrolled through a combination of retrospective collection of information from our hospital outpatient records and prospective screening of patients. The enrolled patients were prospectively followed up at 30 and 60 days after initial outpatient treatment. (Fig. 1).

The inclusion criteria were as follows: (1) COVID-19 infection diagnosed using the novel coronavirus antigen or polymerase chain reaction, (2) cough lasting for more than 8 weeks after COVID-19 infection, and (3) chest imaging showed no significant abnormalities. The exclusion criteria were as follows: (1) chronic cough caused by bronchiectasis, chronic obstructive pulmonary disease, asthma or other basic lung diseases (assessed by outpatient physicians based on past history and family history), (2) presence of uncontrolled malignant tumors, (3) age < 14 years old, and (4) had received oral or parenteral corticosteroids or ICS within one month before the COVID-19 infection (determined by prescription documentation in the medical records and the patients’ self-reported history of corticosteroids medication use). The detailed clinical data of eligible patients were recorded, including basic information, medical history, diagnosis, and pulmonary function test results. All eligible patients received conventional symptomatic cough suppressants (compound methoxyphenamine and cough syrup) and/or expectorant medications, and some also received additional ICS therapy (budesonide/formoterol powder for inhalation 2 puffs per day). ICS was initially prescribed according to the following criteria: (1) patients experiencing dyspnea, wheezing, or irritating cough with a positive bronchial provocation test result; (2) patients with nighttime symptoms; (3) patients who reported poor efficacy of previous self-administered cough medications. Prescriptions were written by four experienced clinicians. Patients who did not use ICS as prescribed and those without ICS prescriptions were included in the non-ICS (NICS) group, while patients who used ICS were assigned to the ICS group.

Verbal informed consent was obtained from all patients who were willing to participate in this study during the telephone surveys, and those who refused to participate were excluded. This study was approved by the Ethics Committee of the Fifth Medical Center of the Chinese PLA General Hospital (approval number: KY-2023-6-44-1).

### Follow-up assessment of the participants

All participants received telephone follow-up, and willing patients attended face-to-face interviews at the outpatient service of our hospital 1 and 2 months after the initial outpatient visit. The cough evaluation test (CET), modified British Medical Research Council (mMRC) dyspnea scale score, and pulmonary function test were conducted. Fractional exhaled nitric oxide (FeNO) and concentration of alveolar nitric oxide (CaNO) measurements were recorded. Specific interviews and identical questionnaires, including the CET and mMRC dyspnea scale, were conducted by one trained researcher in the telephone follow-up surveys and by four clinicians in the face-to-face follow-up interviews to maintain a record of the clinical data for both groups.

Cough severity was assessed using the CET, which includes five items: degree of daytime cough, effect of nighttime cough on sleep, intensity of cough, and effect of cough on daily life and psychology. Scores ranged from 0 to 25, with 0 indicating no cough and a higher score indicating higher cough severity [13, 14].

The mMRC dyspnea scale was used to assess the severity of dyspnea in patients. It comprises five grades (0–4), which are scored according to the degree of activity tolerance in patients with shortness of breath, with level 4 indicating difficulty breathing when undertaking the slightest activity [15].

Spirometry, measurement of exhaled lower respiratory nitric oxide and diffusing capacity of the lungs for carbon monoxide were measured using established norms [16–18]. A FeNO_50_ value of ≥ 25 ppb indicated large airway inflammation, a FeNO_200_ value of > 10 ppb suggested inflammation of the small airways, and a CaNO value of > 5 ppb indicated alveolar inflammation [19–21].

Asthma was diagnosed in patients with a history of typical symptom patterns (wheezing, shortness of breath, chest tightness, cough) and evidence of variable expiratory airflow limitation, based on the GINA 2023 report [22].

### Outcomes

The primary outcomes were improvement in clinical symptoms, as measured by the CET score, mMRC dyspnea scale score, and symptoms of sequelae reported after 1 and 2 months of initial outpatient treatment, which were assessed in both groups participating in the telephone follow-up survey. Recovery was defined as a complete absence of all symptoms on day 60, whereas no reduction in the symptom scores at the two telephone follow-ups indicated no improvement. The secondary outcomes included changes in spirometry, FeNO, and CaNO, which were tested in patients who attended face-to-face visits after 2 months.

### Statistical analysis

Demographic characteristics and clinical parameters at enrollment were described as absolute values along with percentages for categorical variables. Numerical variables were expressed as the mean ± standard deviation if they conformed to a normal distribution, and the median (interquartile range, IQR) was used for non-normal parameters. Chi-squared test, two-sample t-test, or Mann–Whitney U test was used to compare baseline demographic and clinical parameters between the two groups. The Wilcoxon matched-pairs signed-rank test or paired t-test were used to compare the clinical parameters at baseline to those in the different follow-up visits. The missing data were not imputed. All statistical analyses were performed using IBM SPSS Statistics version 25.0. Statistical significance was defined as a two-sided p-value of < 0.05.

## Results

### Cohort clinical features

**Fig. 2:**
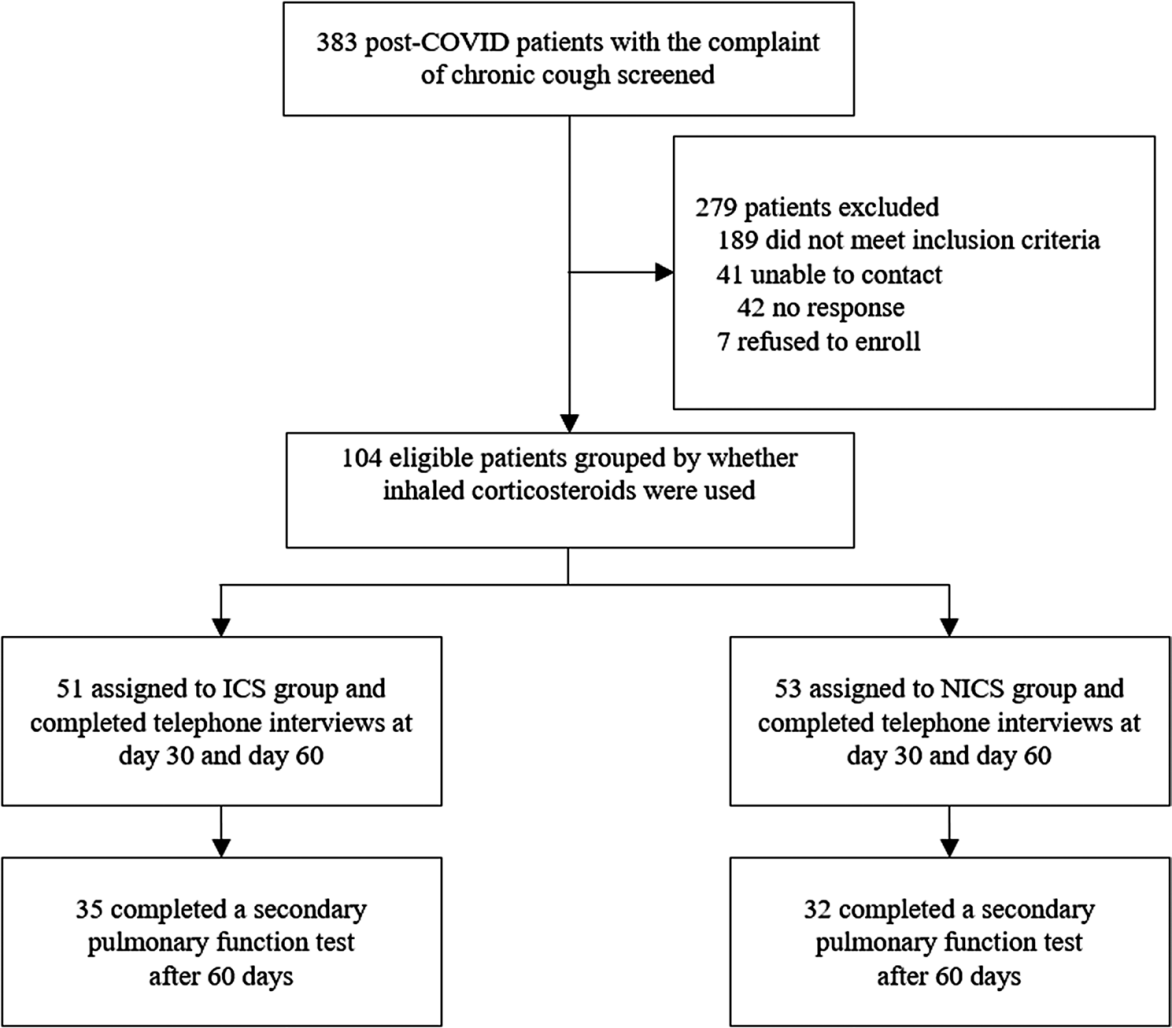
Flow chart of the study. All enrolled patients provided at least one primary outcome. COVID: coronavirus disease; ICS group: inhaled corticosteroid treatment group; NICS group: non-inhaled corticosteroid treatment group

A total of 104 eligible patients were enrolled in this ambispective cohort study. Participants were divided into the ICS (*n* = 51) and NICS (*n* = 53) groups based on the treatment regimens (Fig. 2). The enrolled patients had a median age of 42.5 (35.0–57.3) years and comprised 65 (62.5%) women and 39 (37.5%) men. The most common symptoms accompanying post-COVID-19 chronic cough were sputum (58.7%) and dyspnea (48.1%) (Table 1). There were no significant differences between the two groups in terms of baseline demographics, cough duration, comorbidities, symptoms, CET scores, or mMRC dyspnea scale scores (Table 1).

**Table 1:** Demographic and baseline characteristics of the patients at enrollment

	All patients	ICS group	NICS group	p value
	*N* = 104	*n* = 51	*n* = 53	
Age, years	42.5 (35.0–57.3)	42.0 (38.0–55.0)	44.0 (31.5–60.5)	0.509
Cough duration, weeks	9.5 ± 1.3	9.6 ± 1.4	9.4 ± 1.2	0.458
Sex				0.649
Male	39 (37.5%)	18 (35.3%)	21 (39.6%)	
Female	65 (62.5%)	33 (64.7%)	32 (60.4%)	
Smoking history				
Former smoker	5 (4.8%)	3 (5.9%)	2 (3.8%)	0.965
Current smoker	3 (2.9%)	1 (2.0%)	2 (3.8%)	1.000
Never smoked	96 (92.3%)	47 (92.2%)	49 (92.4%)	1.000
Comorbidity				
Hypertension	17 (16.3%)	6 (11.8%)	11 (20.8%)	0.215
Diabetes	3 (2.9%)	1 (2.0%)	2 (3.8%)	1.000
Cardiovascular and cerebrovascular diseases	3 (2.9%)	0 (0.0%)	3 (5.7%)	0.255
Symptoms				
Dry cough	43 (41.3%)	19 (37.3%)	24 (45.3%)	0.406
Sputum	61 (58.7%)	32 (62.7%)	29 (54.7%)	0.406
Dyspnea	50 (48.1%)	28 (54.9%)	22 (41.5%)	0.172
Wheeze	17 (18.3%)	10 (19.6%)	7 (13.2%)	0.378
Insomnia	4 (3.8%)	1 (2.0%)	3 (5.7%)	0.638
Fatigue	10 (9.6%)	6 (11.8%)	4 (7.5%)	0.692
Dizziness and headache	5 (4.8%)	3 (5.9%)	2 (3.8%)	0.965
Chest pain	5 (4.8%)	3 (5.9%)	2 (3.8%)	0.965
CET	11.0 (9.0–15.0)	12.0 (9.0–16.0)	10.0 (9.0–13.0)	0.142
Dyspnea mMRC				
0	54 (51.9%)	23 (45.1%)	31 (58.5%)	0.172
1 2	27 (26.0%) 14 (13.5%)	15 (29.4%) 7 (13.7%)	12 (22.6%) 7 (13.2%)	0.431 0.938
3	9 (8.7%)	6 (11.8%)	3 (5.7%)	0.448

Age and values of CET are expressed as median (IQR). Sex, smoking history, comorbidity, symptoms, mMRC dyspnea scale, and bronchial provocation test are expressed as frequency (percentage)

CET: cough evaluation test; mMRC: modified British Medical Research Council dyspnea scale. ICS: inhaled corticosteroid group; NICS: non-inhaled corticosteroid group. P value: ICS group versus NICS group

**Fig. 3:**
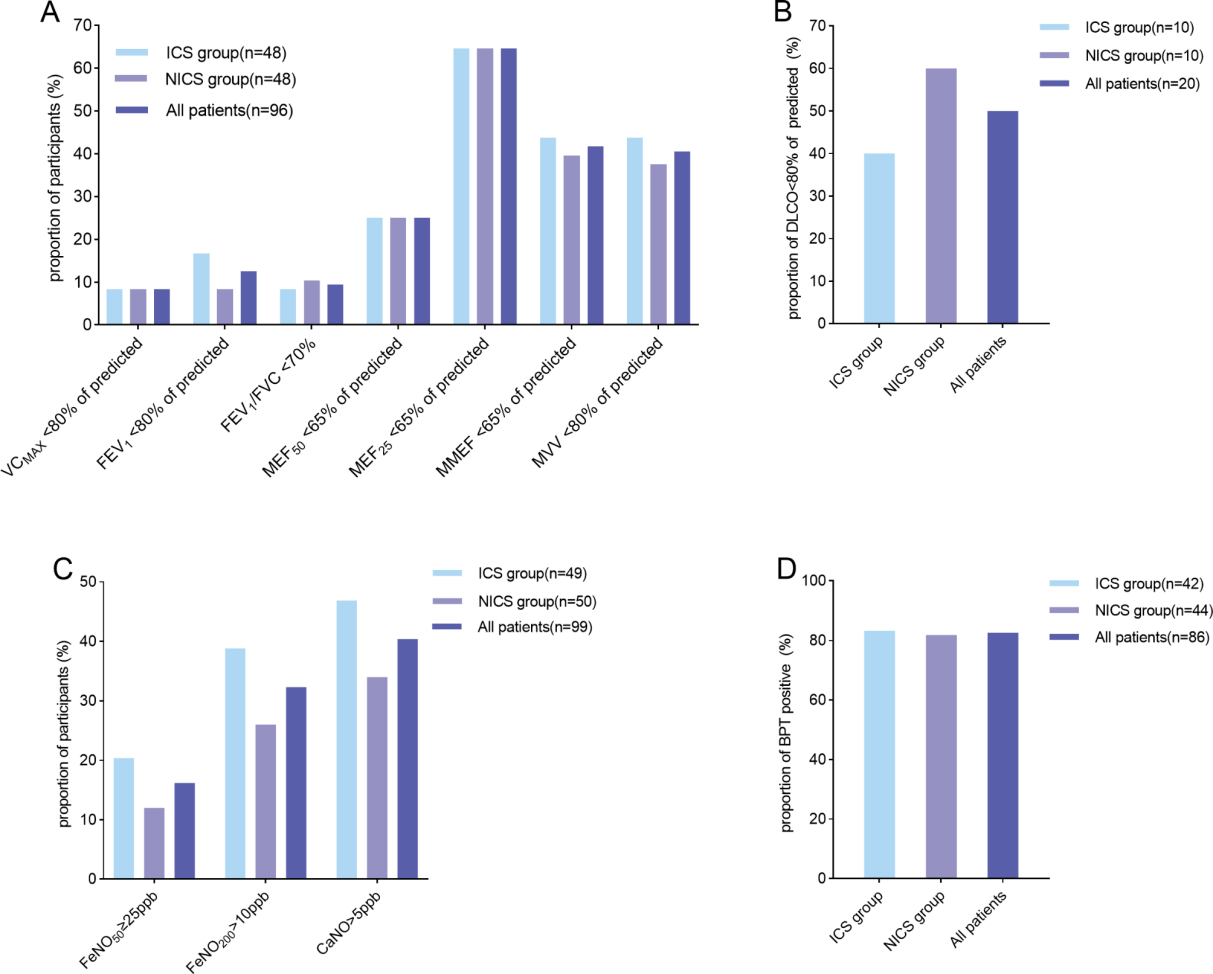
Proportion of patients with abnormal lung examination parameters at enrollment. Proportion of patients with abnormal pulmonary function test parameters (**A**); DLCO < 80% of predicted (**B**); abnormal exhaled NO level (**C**); positive results for the bronchial provocation test (**D**). VC: vital capacity; FVC: forced vital capacity; FEV_1_: forced expiratory volume in the first second; MEF_50_: maximal expiratory flow after 50% of the FVC has been not exhaled; MEF_25_: maximal expiratory flow after 25% of the FVC has not been exhaled; MMEF: maximal mid-expiratory flow; MVV: maximal ventilatory volume; NO: nitric oxide; DLCO: diffusion capacity for carbon monoxide; FeNO_50_: exhaled NO at a flow rate of 50 mL/s; FeNO_200_: exhaled NO at a flow rate of 200 mL/s; CaNO: concentration of alveolar NO; BPT: bronchial provocation test; ICS: inhaled corticosteroid group; NICS: non-inhaled corticosteroid group

The spirometry test, exhaled nitric oxide test, and bronchial provocation test findings of the participants are shown in Fig. 3. Forty (41.7%) patients had small airway dysfunction, 18 (18.8%) were diagnosed with obstructive pulmonary ventilation dysfunction, and 6 (6.3%) had restrictive pulmonary dysfunction. The difference in the proportion of abnormal lung function between the two groups was not statistically significant (all *p* > 0.05; Fig. 3A). Ten (50.0%) patients had decreased lung diffusion function (Fig. 3B). As shown in Fig. 3C, a FeNO_50_ value of ≥ 25 ppb was detected in 16 (16.2%) patients, a FeNO_200_ value of > 10 ppb was detected in 32 (32.3%) patients, and a CaNO value of > 5 ppb was detected in 39 (39.4%) patients. The two groups exhibited comparable FeNO and CaNO levels. Furthermore, 71 (82.6%) patients showed positive results in the bronchial provocation test (Fig. 3D), and 49 (47.1%) patients were diagnosed with asthma.

### Changes in symptoms, cough and dyspnea

We compared the presence of self-reported symptoms before and after treatment with ICS. The results are shown in Table 2. Sixty-seven (64.4%) patients reported recovery and 32 (30.8%) patients reported improved symptoms. However, 5 patients showed no symptom improvement. In addition, compared to baseline, the severity of cough was significantly reduced on days 30 and 60 in both groups (*p* < 0.001). The median change in the CET scores in the ICS group was greater than that in the NICS group; however, there was no statistically significant difference in the change between the two groups (day 30 change from baseline, *P* = 0.178; day 60 change from baseline, *p* = 0.094) (Table 2). In terms of breathing difficulty, the ICS group showed obvious improvement in the mMRC score, with a median of 1.0 (IQR: 0.0–2.0) at baseline and 0.0 (0.0–0.0) at day 60 (*p* < 0.001). Likewise, the NICS group showed a similar change at follow-up, with a median mMRC score of 0.0 (IQR: 0.0–1.0) at baseline and 0.0 (0.0–0.0) at day 60 (*p* < 0.001). The dyspnea scale scores between the two groups at each visit and the median change from baseline to day 60 were comparable (all *p* > 0.05) (Table 2). Similar results were seen in the subgroup analyses of the 49 patients with newly diagnosed asthma (See Supplementary Table 1, Additional File 1). Significant improvements in cough and dyspnea symptoms were observed in patients with asthma whether or not they received ICS.

**Table 2:** Primary outcomes after 30 days and 60 days

	ICS group (*n* = 51)	NICS group (*n* = 53)	p value
Symptoms after 60 days			
Recovery	31 (60.8%)	36 (67.9%)	0.447
Improved	18 (35.3%)	14 (26.4%)	0.327
Not improved	2 (3.9%)	3 (5.7%)	1.000
CET			
Baseline, median (IQR)	12.0 (9.0, 16.0)	10.0 (9.0, 13.0)	0.142
Day 30, median (IQR)	7.0 (6.0, 10.0) ^a^	6.0 (5.5, 7.5) ^a^	0.101
Day 60, median (IQR)	5.0 (5.0, 6.0) ^a^	5.0 (5.0, 6.0) ^a^	0.578
Day 30 change from baseline, median (IQR)	−5.0 (− 7.0, − 2.0)	−3.0 (− 5.0, − 2.0)	0.178
Day 60 change from baseline, median (IQR)	−7.0 (− 10.0, − 3.0)	−5.0 (− 7.0, − 3.0)	0.094
mMRC dyspnea score			
Baseline, median (IQR)	1.0 (0.0, 2.0)	0.0 (0.0, 1.0)	0.167
Day 30, median (IQR)	0.0 (0.0, 1.0) ^a^	0.0 (0.0, 1.0) ^a^	0.511
Day 60, median (IQR)	0.0 (0.0, 0.0) ^a^	0.0 (0.0, 0.0) ^a^	0.361
Day 30 change from baseline, median (IQR)	0.0 (− 1.0, 0.0)	0.0 (− 1.0, 0.0)	0.200
Day 60 change from baseline, median (IQR)	0.0 (− 1.0, 0.0)	0.0 (− 1.0, 0.0)	0.218

Symptom conditions at day 60 are presented as frequency (percentage). Values of CET, mMRC score, and change values are presented as median (IQR). CET: Cough evaluation test; IQR: interquartile range; mMRC: modified British Medical Research Council dyspnea scale; ICS: inhaled corticosteroid; NICS: non-inhaled corticosteroid. P value: ICS group versus NICS group. Compare to baseline: ^a^*p* < 0.001

### Secondary outcomes

A second examination was performed 2 months after the initial visit in 67 patients, of whom 35 were in the ICS group and 32 were in the NICS group. Table 3 shows that in the ICS group, the maximum vital capacity (VC_MAX_)%, forced expiratory volume in the first second (FEV_1_) %, maximal mid-expiratory flow (MMEF)% and maximal ventilatory volume (MVV)% of predicted, FEV_1_/FVC, and FeNO_50_ levels were significantly improved from the baseline levels. In the NICS group, a measurable increase in the MVV% of the predicted relative to pretreatment was observed. Additionally, the changes in the FEV_1_%, MMEF%, and MVV% of predicted, FEV_1_/FVC, and the FeNO_50_ levels showed obvious differences between the two groups. The results of the bronchial provocation tests showed that 12 (41.4%) patients changed from positive to negative in the ICS group, which was more than that observed in the NICS group (23.1%); however, the difference was not statistically significant. The comparison of the VC_MAX_%, FEV_1_%, MMEF%, MVV% of predicted, FEV_1_/FVC, and the FeNO_50_ levels between the two visits showed significant differences in the subgroup analysis of patients with newly diagnosed asthma using ICS (See Supplementary Table 2, Additional File 1). Moreover, the changes in the MMEF% of predicted and FEV_1_/FVC were significantly different between the groups of patients with newly diagnosed asthma receiving ICS and those not receiving ICS.

**Table 3:** Change in pulmonary function after 60 days

		ICS group	NICS group	p value
		*n* = 35	*n* = 32	
VC_MAX_%	Baseline	94.7 ± 10.0	94.8 ± 11.5	0.955
	Day 60	98.0 ± 10.7	96.1 ± 11.0	0.475
		(p’ = 0.001) ^b^	(p’ = 0.123)	
	Change	2.3 (− 1.1, 5.9)	0.5 (− 1.1, 4.4)	0.192
FEV_1_%	Baseline	91.4 ± 11.2	91.2 ± 12.4	0.941
	Day 60	95.9 ± 11.9	92.6 ± 12.5	0.269
		(p’ = 0.001) ^b^	(p’ = 0.068)	
	Change	3.9 (1.5, 6.9)	1.7 (− 0.4, 3.1)	0.007^a^
FEV_1_/FVC	Baseline	79.5 ± 5.9	80.0 ± 7.9	0.773
	Day 60	81.4 ± 5.4	79.8 ± 6.7	0.284
		(p’ < 0.001) ^b^	(p’ = 0.721)	
	Change	1.6 (0.5, 3.1)	0.8 (− 1.5, 1.3)	0.001^a^
MMEF%	Baseline	68.4 ± 19.5	68.9 ± 22.8	0.928
	Day 60	77.6 ± 18.1	70.6 ± 22.4	0.163
		(p’ < 0.001) ^b^	(p’ = 0.096)	
	Change	9.8 (3.8, 12.0)	2.2 (− 1.9, 5.8)	0.000^a^
MVV %	Baseline	82.3 ± 16.7	82.2 ± 14.9	0.996
	Day 60	92.9 ± 17.4	87.9 ± 16.9	0.230
		(p’ < 0.001) ^b^	(p’ = 0.001) ^b^	
	Change	10.7 (4.1, 16.9)	3.5 (1.8, 9.2)	0.013^a^
		***n*** **= 34**	***n*** **= 31**	
FeNO_50_, ppb	Baseline	19.5 (13.5, 26.5)	15.0 (12.0, 22.0)	0.161
	Day 60	15.5 (11.8, 21.5)	14.0 (12.0, 21.0)	0.843
		(p’ < 0.001) ^b^	(p’ = 0.215)	
	Change	−3.0 (− 7.0, − 0.0)	−1.0 (− 2.8, 1.0)	0.041^a^
FeNO_200_, ppb	Baseline	8.5 (6.0, 11.5)	8.0 (7.0, 12.0)	0.974
	Day 60	7.5 (7.0, 11.5)	9.0 (6.0, 11.0)	0.521
		(p’ = 0.100)	(p’ = 0.280)	
	Change	−1.0 (− 4.0, 1.3)	0.0 (− 2.8, 1.8)	0.647
CaNO, ppb	Baseline	4.1 (1.9, 5.3)	4.8 (2.7, 5.0)	0.482
	Day 60	3.3 (2.8, 4.3)	4.0 (3.1, 4.8)	0.229
		(p’ = 0.064)	(p’ = 0.098)	
	Change	−1.1 (-2.2, 0.8)	−0.5 (− 2.3, 0.8)	0.857
**BPT**		***n*** **= 30**	***n*** **= 30**	
Positive	Baseline	29 (96.7%)	26 (86.7%)	0.350
	Day 60	17 (56.7%)	20 (66.7%)	0.302
		(p’ < 0.001) ^b^	(p’ = 0.067)	
	Change	12 (41.4%)	6 (23.1%)	0.149

VC_MAX_%, MMEF%, and MVV% of predicted and FEV_1_/FVC in two visits are expressed as mean ± standard deviation. The FeNO_50_ value and change between two visits are expressed as median (interquartile range). Bronchial provocation test is expressed as frequency (percentage). VC: vital capacity; FVC: forced vital capacity; FEV_1_: forced expiratory volume in the first second; MMEF: maximal mid-expiratory flow; MVV: maximal ventilatory volume; NO: nitric oxide; FeNO_50_: exhaled NO at a flow rate of 50 mL/s; FeNO_200_: exhaled NO at a flow rate of 200 mL/s; CaNO: concentration of alveolar NO; BPT: bronchial provocation test ICS: inhaled corticosteroid group; NICS: non-inhaled corticosteroid group

P value: ICS group versus NICS group; p’: day 60 versus baseline. ^a^ Significant difference between ICS group and NICS group; ^b^ Significant difference between day 60 and baseline

## Discussion

In the current study, we first explored the efficacy of ICS therapy in patients with post-COVID-19 chronic cough. We found that chronic cough after COVID-19 was mostly of moderate severity and accompanied by mild dyspnea symptoms and airway hyperresponsiveness, with some patients showing abnormal parameters on pulmonary function tests. In addition, we also found that a surprisingly high number (over 40%) of patients developed asthma after COVID-19 infection. In our follow-up, most patients showed significant improvements in symptoms regardless of whether ICS was used. However, lung function was significantly improved in patients receiving ICS treatment.

The demographic analysis revealed that the majority of patients with chronic cough after COVID-19 were female (65, 62.5%), which may be related to the research conclusion of Jassat et al. that women are more susceptible to the onset of COVID-19 [23]. However, a retrospective cohort study on the incidence of long COVID during the Omicron wave in eastern India found that sex was not a significant predictor of long COVID [24]. In addition, the prevalence of non-COVID-19 chronic cough has been reported to be higher among women, which may be due to higher cough reflex sensitivity [25]. These findings suggest that the higher prevalence of post-COVID-19 chronic cough in female patients may be due to certain innate physiological characteristics; however, this has not been investigated in previous studies.

We observed that post-COVID-19 chronic cough during the Omicron era frequently co-occurred with dyspnea and airway hyperresponsiveness, and the observed elevated FeNO levels also suggested a possible association with Th2 inflammation. Although we did not set up a non-COVID chronic cough control group, a previous study showed that post-COVID chronic cough is more likely to be accompanied by dyspnea [26]. Compared to our results, a higher proportion (nearly half) of the patients with chronic cough in the cohort in that study had elevated FeNO levels [26]. We speculate that this may be because patients with pre-existing asthma were not excluded from the cohort, and exhaled nitric oxide levels are elevated in patients with asthma [27]. Extensive airway inflammation and respiratory epithelial damage caused by viral infection are the direct intrinsic triggers of post-infectious cough, while airway hyperresponsiveness and cough reflex hypersensitivity aggravate the symptoms [28]. Although comprehensive testing has not been conducted to verify the possible pathogenesis, we believe that the pathogenesis of post-COVID chronic cough may not be exceptional compared to other post-infectious coughs.

We also found that post-COVID-19 chronic cough was accompanied by lung dysfunction, as some patients had obstructive or restrictive pulmonary ventilation dysfunction, and approximately half had abnormal minor airway function. Previous studies have reported respiratory symptoms and pulmonary dysfunction in patients with COVID-19, where similar abnormalities in pulmonary function test parameters were observed [29–31]. This suggests the importance of pulmonary function tests for patients with persistent cough after COVID-19. Post-COVID-19 persistent cough is commonly accompanied by dyspnea, polypnea, weakness, muscular soreness, or other multisystem manifestations, which may indicate complex multifactor pathogenesis [9]. Therefore, recording the accompanying symptoms and their degree can contribute to the identification and follow-up of patients with persistent cough.

Respiratory viruses can aggravate or induce asthma. The COVID-19 pandemic has increased the attention of researchers to patients with asthma. Current research results show that existing asthma does not seem to increase a patient’s susceptibility to SARS-CoV-2 infection or affect the severity of the disease [32]. However, some research has supported the idea that SARS-CoV-2 infection can cause an exacerbation of asthma or even contribute to the initial development of a clinical asthma attack [33, 34]. Our study provides evidence to support this perspective; more than 40% of patients with persistent symptoms of cough or breathlessness after COVID-19 infection were shown to have asthma. This proportion is higher than that reported in a previous study, where approximately one tenth of patients were diagnosed with newly emerging asthma [35]. Our results may show a higher rate because positive bronchial provocation test results caused by transient airway hyperresponsiveness may increase the diagnosis of cough variant asthma.

ICS is the strongest local airway anti-inflammatory drug commonly used to control asthma symptoms and improve lung function. Inhaled budesonide can regulate inflammation by reducing epithelial damage and improving the T cell response, and that early use of ICS therapy can improve the inflammatory manifestations of patients with COVID-19 in the acute phase, producing clinical benefits [36–38]. Airway inflammation is a central mechanism in post-infectious cough; therefore, ICS may be an effective option for patients who develop a persistent cough after COVID-19 infection. The results of our study showed that most patients exhibited a substantial return to their pre-COVID-19 status at the 2-month follow-up, with significant reductions in CET and dyspnea scores. Nevertheless, consistent with other studies [26, 39], a minority of patients showed no improvement after treatment. Moreover, the level of symptomatic relief was comparable in patients with post-COVID-19 chronic cough, regardless of whether corticosteroids were added to the treatment schedule. While greater changes in CET scores were found in patients treated with ICS, the differences were not statistically significant. In terms of improvement in lung function, in the analyses of the pulmonary function tests at the two visits, the patients treated with ICS showed a more significant improvement in lung function compared to those treated with conventional therapy; however, a very small number of patients showed no improvement or even deterioration of lung function. In a 2-year follow-up study, the lung function parameters of patients hospitalized due to COVID-19 of different severities showed no improvement after discharge [40]. This discrepancy with our results may be because all of our participants were outpatients with mild lung function impairment. Therefore, our study shows that the effect of ICS on patients with chronic cough after COVID-19 may be greater than that observed with non-inhalation therapy.

This study had several limitations. First, this was a single-center study with a small sample size; therefore, the reproducibility and external validity of the results may be limited. In addition, the subjects were recruited from respiratory clinics; therefore, it may not be possible to generalize the results to hospitalized or severely ill patients. Second, without a non-COVID-19 chronic cough group as a control, it is difficult to determine whether the observed features are distinct for patients with post-COVID-19 chronic cough. Third, the response rate of patients could cause selection bias. However, the baseline characteristics of patients in the ICS and the NICS group were balanced. Patients who did not participate may have milder symptoms than those who did, potentially leading to an overestimation of the rate of asthma diagnosis. Forth, the use of ICS is affected by the choice of patients. It is possible that some patients who respond to ICS treatment did not use ICS, which might result in an underestimation of the effectiveness of ICS. Fifth, several parameters were not collected in the follow-up, including the specific time taken for symptoms to improve and further details of medication adherence and combination medication. However, our study may reflect a real-world experience. Finally, given the self-limited nature of post-infectious cough, the symptom improvements in our cohort may be partly attributed to spontaneous remission. Thus, a longer follow-up period is necessary to verify our results over time.

Despite the limitations, we focused on treatment options for patients with persistent cough following COVID-19 infection during the Omicron wave, which has not been previously reported.

## Conclusion

In conclusion, chronic cough caused by infection with the Omicron variant is often accompanied by dyspnea. Most patients had airway hyperresponsiveness, and there was an asthma prevalence of 47.1% in the cohort. The potential cause of persistent cough after COVID-19 may be viral infection related-asthma. While the effect of ICS on symptom improvement may not be superior to that of conventional treatment, ICS therapy can improve lung function in patients with post-COVID-19 chronic cough. Further studies are required to evaluate the long-term improvements in patients undergoing ICS therapy.

### Electronic supplementary material

Below is the link to the electronic supplementary material.

Changes in CET, mMRC and pulmonary function in newly diagnosed asthma patients

## Data Availability

Restrictions apply to the availability of these data and they are not publicly available. However, data are available from the corresponding author upon reasonable request and with the permission of the institution.

## Abbreviations

CaNO: concentration of alveolar nitric oxide
CET: cough evaluation test
COVID-19: coronavirus disease 2019
FeNO: fractional exhaled nitric oxide
FEV_1_: forced expiratory volume in the first second
FVC: forced vital capacity
ICS: inhaled corticosteroid
IQR: interquartile range
mMRC: modified British Medical Research Council
MMEF: maximal mid-expiratory flow
MVV: maximal ventilatory volume
NICS: non-inhaled corticosteroid
SARS-CoV-2: severe acute respiratory syndrome coronavirus 2
