# Supplementary material for: Clinical characteristics and effects of inhaled corticosteroid in patients with post-COVID-19 chronic cough during the Omicron variant outbreak
Source: BMC Pulm Med. 2024 Mar 27;24:156. doi: 10.1186/s12890-024-02937-7 (PMC10967081; doi:10.1186/s12890-024-02937-7)
Supplement: Supplementary file 1 — Changes in CET, mMRC and pulmonary function in newly diagnosed asthma patients [file 12890_2024_2937_MOESM1_ESM.docx]

**Clinical characteristics and effects of inhaled corticosteroid in patients with post-COVID-19 chronic cough during the Omicron variant outbreak**

Pan-Pan Xie^1, 2, 4^ ^#^; Yue Zhang^2,^ ^#^; Wen-Kai Niu^2, #^; Bo Tu^2^; Ning Yang^2^; Yun Fang^2^; Ying-Hui Shi^2^; Fu-Sheng Wang^3,^ *; Xin Yuan^1, 2,^ *

^1^ Anhui Medical University PLA 307 Clinical College, Beijing, China;

^2^ Department of Respiratory and Critical Care Diseases, Department of Infectious Diseases, the Fifth Medical Center of Chinese PLA General Hospital, Beijing, China;

^3^ Department of Infectious Diseases, Fifth Medical Center of Chinese PLA General Hospital, National Clinical Research Center for Infectious Diseases, Beijing, China;

^4^ The Fifth Clinical Medical College of Anhui Medical University, Hefei, Anhui, China.

^#^Pan-Pan Xie, Yue Zhang and Wen-Kai Niu have contributed equally to this work.

**Corresponding authors**

*Correspondence to Xin Yuan (xinyuannatile@sina.com, Department of Respiratory and Critical Care Diseases, Department of Infectious Diseases, Fifth Medical Center of Chinese PLA General Hospital, Beijing, China) or Fu-Sheng Wang (fswang302@163.com, Department of Infectious Diseases, Fifth Medical Center of Chinese PLA General Hospital, National Clinical Research Center for Infectious Diseases, Beijing, China).

**Supplementary Table 1** CET and mMRC change in newly diagnosed asthma patients after 30 and 60 days

|  | **AICS group (*n* = 28)** | **ANICS group (*n* = 21)** | **p value** |
| --- | --- | --- | --- |
| CET  Baseline, median (IQR)  Day 30, median (IQR)  Day 60, median (IQR)  Day 30 change from baseline, median (IQR)  Day 60 change from baseline, median (IQR)  mMRC dyspnea score  Baseline, median (IQR)  Day 30, median (IQR)  Day 60, median (IQR)  Day 30 change from baseline, median (IQR)  Day 60 change from baseline, median (IQR) | 13.5 (9.25, 17.75)  7.0 (6.0, 10.0) ^a^  5.0 (5.0, 6.0) ^a^  −5.0 (−7.75, −2.0)  −6.5 (-11.5, −4.25)  0.0 (0.0, 1.0)  0.0 (0.0, 1.0) ^b^  0.0 (0.0, 0.0) ^b^  0.0 (−0.75, 0.0)  0.0 (−1.0, 0.0) | 11.0 (9.0, 14.0)  7.0 (6.0, 8.0) ^a^  5.0 (5.0, 8.0) ^a^  −4.0 (−7.0, −1.5)  −6.0 (−8.0, −3.0)  0.0 (0.0, 2.0)  0.0 (0.0, 1.0) ^b^  0.0 (0.0, 0.0) ^b^  0.0 (−1.0, 0.0)  0.0 (−2.0, 0.0) | 0.330  0.550  0.717  0.290  0.201  0.830  0.639  0.102  0.584  0.656 |

Values of CET, mMRC score, and change values are presented as median (IQR). CET: Cough evaluation test; IQR: interquartile range; mMRC: modified British Medical Research Council dyspnea scale; AICS: asthma patients using inhaled corticosteroid; ANICS: asthma patients not using inhaled corticosteroid. *P* value: AICS group versus ANICS group. Compare to baseline: ^a^ p < 0.001, ^b^ p < 0.05.

**Supplementary Table 2** Changes in pulmonary function in newly diagnosed asthma patients after 60 days.

|  |  | **AICS group** | **ANICS group** | **p value** |
| --- | --- | --- | --- | --- |
| VC_MAX_% of predicted  FEV_1_ % of predicted  FEV_1_/FVC  MMEF% of predicted  MVV % of predicted  **Exhaled nitric oxide**  FeNO_50_, ppb  FeNO_200_, ppb  CaNO, ppb  **Bronchial provocation test**  Positive | Baseline  Day 60  Change  Baseline  Day 60  Change  Baseline  Day 60  Change  Baseline  Day 60  Change  Baseline  Day 60  Change  Baseline  Day 60  Change  Baseline  Day 60  Change  Baseline  Day 60  Change  Baseline  Day 60  Change | ***n* = 25**  94.8±9.8  97.5±10.9 (p’ = 0.034) ^b^  0.9 (−2.0, 5.2)  92.6±10.7  96.3±12.0 (p’ = 0.030) ^b^  3.1 (1.0, 4.6)  80.4±5.6  82.2±5.3 (p’ = 0.001) ^b^  1.6 (0.3, 2.6)  70.3±19.7  78.8±19.1 (p’ < 0.001) ^b^  7.3 (3.5, 13.6)  82.9±17.8  93.2±18.5 (p’ < 0.001) ^b^  7.6 (3.9, 14.0)  ***n* = 24**  22.5 (12.0, 32.5)  16.5 (11.3, 26.0) (p’ = 0.001) ^b^  −4.0 (−7.5, 0.0)  8.5 (6.3, 12.5)  8.0 (7.0, 11.3) (p’ = 0.199)  −1.0 (−3.8, 1.0)  3.9 (1.5, 5.1)  2.8 (1.9, 4.1) (p’ = 0.072)  −1.3 (−2.5, 0.9)  ***n* = 22**  22 (100.0%)  14 (63.7%) (p’ = 0.006) ^b^  8 (36.4%) | ***n* = 20**  94.1±11.7  94.7±9.1 (p’ = 0.597)  0.5 (−1.4, 3.8)  90.1±12.5  90.7±9.8 (p’ = 0.441)  1.7 (−2.3, 2.5)  80.1±8.2  80.4±7.5 (p’ = 0.189)  0.8 (−0.7, 1.3)  68.3±25.0  71.3±24.1 (p’ = 0.017) ^b^  2.2 (−1.0, 6.0)  84.1±15.9  89.3±17.9 (p’ = 0.016) ^b^  2.8 (1.3, 9.4)  ***n* = 19**  17.0 (12.0, 22.0)  16.0 (12.0, 21.0) (p’ = 0.444)  0.0 (−3.8, 1.8)  9.0 (7.0, 13.0)  9.0 (7.0, 11.0) (p’ = 0.154)  0.0 (−6.0, 1.0)  4.8 (2.7, 5.6)  4.0 (3.1, 4.8) (p’ = 0.112)  −0.4 (−2.8, 0.4)  ***n* = 18**  18 (100.0%)  14 (77.8%) (p’ = 0.112)  4 (22.2%) | 0.830  0.348  0.486  0.463  0.101  0.051  0.868  0.367  0.003^a^  0.763  0.250  0.013^a^  0.809  0.485  0.054  0.174  0.863  0.095  0.530  0.374  0.840  0.419  0.078  0.689  NA  0.332  0.332 |

VC_MAX_%, MMEF%, and MVV% of predicted and FEV_1_/FVC in two visits are expressed as mean ± standard deviation.

The FeNO_50_ value and change between two visits are expressed as median (interquartile range).

Bronchial provocation test result is expressed as frequency (percentage).

VC: vital capacity; FVC: forced vital capacity; FEV_1_: forced expiratory volume in the first second; MMEF: maximal mid-expiratory flow; MVV: maximal ventilatory volume; NO: nitric oxide; FeNO_50_: exhaled NO at a flow rate of 50 mL/s; FeNO_200_: exhaled NO at a flow rate of 200 mL/s; CaNO: concentration of alveolar NO. AICS: asthma patients using inhaled corticosteroid; ANICS: asthma patients not using inhaled corticosteroid.

P value: AICS group versus ANICS group; p’: day 60 versus baseline.

^a^ Significant difference between AICS group and ANICS group; ^b^ Significant difference between day 60 and baseline.
